# Scent of death: Emission and behavioral role of 1-nonene in entomopathogenic nematode *Steinernema kraussei*

**DOI:** 10.1371/journal.pone.0328628

**Published:** 2025-07-28

**Authors:** Rasa Čepulytė, Evelina Osinska, Violeta Apšegaitė, Deimantė Tiškevičiūtė, Vincas Būda

**Affiliations:** Nature Research Centre, Laboratory of Chemical and Behavioral Ecology, Vilnius, Lithauania; National Research Centre, EGYPT

## Abstract

Entomopathogenic nematodes (EPNs) provide a natural alternative to synthetic pesticides for pest control. Understanding how EPNs respond to volatile cues including those released by infected cadavers is crucial for determining the behavior control necessary for improving their effectiveness. In this study, we tested the response of *Steinernema kraussei*, a cruiser foraging species, to 1-nonene. Additionally, we analyzed the emission dynamics of this compound from *Galleria mellonella* larvae infected by three different EPN species: *S. kraussei, S. carpocapsae,* and *S. feltiae*. Concentrations of 1-nonene at 20 mM and above elicited a repellent behavior in *S. kraussei*. Ethanol, used as a solvent for 1-nonene, was attractive to this EPN at high concentrations only. The emission of 1-nonene was the lowest from larvae infected by *S. feltiae* compared to those infected by the other two species. Larvae infected with *S. kraussei* released 15 times more 1-nonene than those infected with *S. feltiae*, while larvae infected with *S. carpocapsae* released 76 times more than those infected with *S. feltiae* and 5 times more than those infected with *S. kraussei*. Comparing our findings to previous research behavioral responses to environmental compounds (ethanol and 1-nonene) suggest that EPNs with cruiser (*S. kraussei*) and intermediate (*S. feltiae*) foraging strategies show similar responses, distinct from those of ambusher’s (*S. carpocapsae*).

## Introduction

Entomopathogenic nematodes (EPNs) are commercially available obligatory parasites of insects that are applied as an environmentally friendly tool to control economically important insect pests [1]. Most of the EPNs belong to two families, Heterorhabditidae and Steinernematidae. Third-stage juveniles, also called infective juveniles (IJs), are the only non-feeding free-living stage searching for and invading suitable insect hosts in the soil. IJs enter the insect through natural body cavities and release their symbiotic bacteria into the hemolymph [2]. Steinernematidae EPNs release *Xenorhabdus* bacteria and Heterorhabditidae – *Photorhabdus* [3]. Bacteria kill and degrade the insect and protect the nematodes from other infections by producing antimicrobial compounds [4]. From two to three generations of nematodes feed on the bacteria-digested insect tissues and bacteria until they completely explore the cadaver and form a new generation of IJs, which leave the cadaver and disperse to hunt a new victim [5]. EPN IJs have three main host-seeking strategies: cruisers that actively seek sedentary insect hosts in the soil (e.g., *Steinernema kraussei* (Steiner, 1923) Travassos, 1927); ambushers that sit and wait for the mobile insects (e.g., *S. carpocapsae* (Weiser, 1955) Wouts et al., 1982); and intermediate (e.g., *S. feltiae* (Filipjev, 1934) Wouts et al., 1982), that depending on the environment and the host can switch their strategies between cruiser and ambusher [6].

Among the various sensations (thermoreception, mechanoreception, etc.), nematodes possess chemoreception, which is vital to finding a suitable insect host, a source of feed, and a place for reproduction. A number of chemical compounds are known to manipulate EPN behavior, from CO_2_, which indicates the presence of a biological object, to others, more specific ones released by potential prey (insects), their damaged plants, or even EPN already-infected insect cadavers (reviewed by [7]), which point in the right direction towards the prey. However, compared to nematodes from other ecological groups, such as plant parasitic nematodes, for which over several hundred compounds are known [8], very little is understood about the chemical compounds that influence EPN behavior.

EPN-infected insect cadavers release dozens of chemical compounds with limited overlap between different species [9–11]. Among those alkene 1-nonene, was identified as a common volatile for cadavers of several EPN-insect-infections [9]. Until recently, data about 1-nonene as a signaling molecule in the aboveground environment was obtained. For instance, 1-nonene, which is one of the main constituents of the floral scent of Madagascar ragwort (*Senecio madagascariensis*) [12], attracts the mosquito *Aedes aegypti*. It is also released by maize plants (*Zea mays*) that are attacked by aphids (*Rhopalosiphum padi*), where it functions as a cry-for-help signal by attracting ladybirds (*Harmonia axyridis*) – natural enemies of the aphids [13]. In the belowground 1-nonene evokes behavioural reactions in IJs of EPNs [14]. Significant differences were found when studying the reactions of IJs of two EPN species to this compound. Low and high concentrations of 1-nonene attracted and highly repelled *S. feltiae,* respectively, whereas *S. carpocapsae* was attracted to high concentrations of this compound only [14]. Different responses of the IJs suggest an ecological role of this compound in EPN life. The studied species *S. feltiae* and *S. carpocapsae* differ in foraging strategy; the latter is classified as an ambusher, and the former is intermediate between cruiser and ambusher [15]. Can EPN‘s reactions to the chemical compound released from a decaying insect cadaver be related to the foraging strategy? To get closer to solving the question of 1-nonene’s role in EPN ecology, we have set two tasks in this study. 1. To assess the behavioral reactions to 1-nonene of the characteristic cruiser *S. kraussei* [16]. 2. To determine whether the concentrations of 1-nonene that elicit behavioral responses in EPNs align with the concentration range released during the decay of insect larvae. To achieve the latter task, the dynamics of 1-nonene emissions from larvae infected with different EPN species were analyzed.

## Materials and methods

### Nematodes and *G. mellonella*

The initial stock of *S. kraussei* (Nemasys® L) was purchased from Basf, UK, *S. carpocapsae* from Koppert, The Netherlands, and *S. feltiae* (strain RM-107 [17], GenBank Accession number MW480131)) was provided by Dr. Raquel Campos-Herrera, University of La Rioja, Spain. For nematode propagation, approximately 300 IJs in 500 µL deionized water were applied on the surface of five last instar *Galleria mellonella* (Linnaeus 1758) larvae and incubated at room temperature (21–22 °C) for seven days in the dark. IJs were collected using White traps [18] and stored in vented culture tissue flasks for 20 days at 12 °C. Before the behavioral assay, *S. kraussei* IJs were maintained at room temperature (21–22 °C) for 24 h. The behavior of IJs aged five to 20 days was tested. *Galleria mellonella* was reared and maintained at 21 °C on natural honeybee combs. The last instar larvae were collected and stored at 12 °C until used for nematode culturing and in the assays.

### Chemical compounds

1-Nonene is a volatile compound, however, poorly soluble in water, but well in ethanol, hence the response of *S. kraussei* IJs to ethanol was tested first. Ten times and 100 times dilutions of ethanol (96%, Vilniaus Degtinė, Lithuania) were prepared in deionized water. The initial 1 M concentration of 1-nonene (96%, Sigma Aldrich, USA) was prepared by diluting 172.9 µL of 1-nonene in 827.1 µL of ethanol (ratio 1:4:78). The following 1-nonene concentrations of 500 mM, 200 mM, 20 mM, 2 mM, 200 µM, 20 µM, 2 µM, 0.2 µM, and 0.02 µM were prepared in undiluted ethanol and stored at 4 °C until used. Fresh ethanol and 1-nonene concentrations were prepared every two weeks.

### Chemotaxis assay

**Fig 1:**
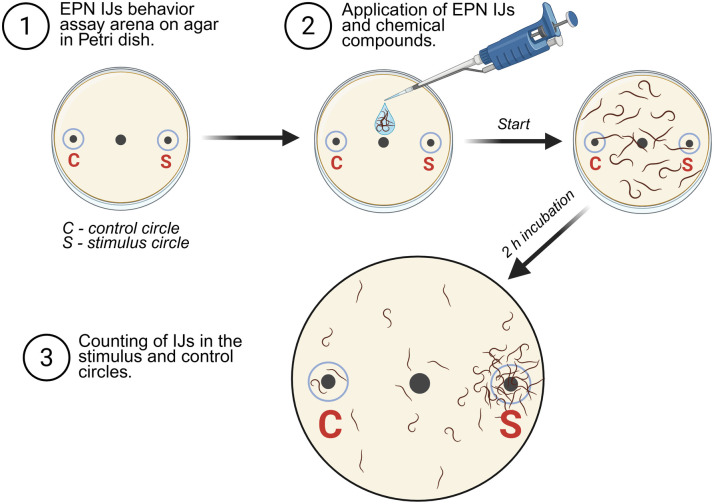
Two-choice chemotaxis assay setup for *Steinernema kraussei* infective juveniles (IJs). Created with BioRender.com.

*Steinernema kraussei* IJs’ two-choice chemotaxis assay was carried out following [14]. Briefly, as IJ behavior assay arenas, 9 cm diam. Petri dishes filled with approximately 15 mL of 1.8% agar (agar-agar, Kobe I, Carl Roth, Germany), were used (Fig 1). A dot in the center of the bottom of a dish was marked as a nematode application point. Two dots 1 cm away from the opposite Petri dish borders were marked as stimulus and solvent (control) application points (Fig 1).

A total of 600 *S. kraussei* IJs in 10 µL of deionized water were applied to the center. Immediately after 10 µL of stimulus, depending on the assay, either ethanol or 1-nonene was applied on one side of the Petri dish, and 10 µL of corresponding control deionized water (solvent of ethanol) or ethanol (solvent of 1-nonene), respectively, was applied on the opposite side. The start of the assay was recorded once the nematode suspension had dried on the agar surface. The dishes were kept at room temperature (21–22 °C) for 2 h in the dark. Immediately after, nematodes that migrated toward the stimulus and control sides were counted using a 1 cm diameter scoring template positioned over the marked dots on the opposite sides of the dish.

Ethanol *vs.* ethanol served as a control for both assays. Two additional controls, positive and neutral, were set to evaluate EPN IJs’ viability and activity, thus ensuring the necessary quality of nematodes used for all the assays. A supernatant of *G. mellonella’s* last instar larva crushed in 300 µL of deionized water *vs.* water served as a positive control, whereas water *vs.* water was a neutral control. Only batches of IJs that were attracted to *G. mellonella* and equally distributed in the water *vs.* water assay were used in further assays. All the experiment controls were applied on agar in the same manner as mentioned above. Each ethanol dilution and each 1-nonene concentration were tested in three Petri dishes simultaneously. All of the assays were conducted six times.

### Sampling of EPN-infected *G. mellonella* larval volatiles

The solid phase micro-extraction (SPME) technique was used to sample the volatiles released by EPN-infected *G. mellonella* larvae in the headspace. For this, ten *G. mellonella* larvae were infected with 200 IJs/larva of either *S. kraussei*, *S. carpocapsae,* or *S. feltiae* (maintained at 12 °C and aged 5–20 days) in the same manner as mentioned above and kept up to 12 days in the dark at room temperature (21–22 °C). The volatiles were collected at 0, 2^nd^, 4^th^, 6^th^, 9^th^, and 12^th^ days after the IJ infection of the larvae. The volatile samplings of each day were replicated 3–5 times. New batches of EPN-infected larvae were prepared for each time point.

For the volatile collection group of ten larvae was transferred to a 15 mL Erlenmeyer glass flask. Volatiles from ten freeze-killed *G. mellonella* larvae and an empty Erlenmeyer glass flask served as controls. The flasks were covered with the foil and kept at 35 °C for 60 min in a water bath to enhance volatility of volatile organic compounds. Before each volatile collection, the routine purification of SPME fiber coated with a polydimethylsiloxane-divinylbenzene polymer (DVB/PDMS, 65 µm coating layer thickness, Supelco, PA, USA) was conducted at 240 ºC for about 10 min in a gas chromatograph’s (GC) injector. Then the needle of the SPME syringe was pierced through the foil into the glass flask just above the EPN-infected larvae, the fiber was pushed out from the needle and exposed to the headspace for 120 min at 35 °C. After the fiber was retracted into the fiber holder and transferred to the injection port of the GC where volatiles were thermally desorbed from the fiber for 2 min.

### Gas chromatography-mass spectrometry analysis

The volatile analyses were performed using a Shimadzu gas chromatograph GC-2010 coupled with a Shimadzu mass spectrometer MS-QP 2010 Plus mass selective detector (Shimadzu, Japan). The GC was equipped with capillary column Rxi® 5Sil MS w/Integra-Guard® (30 m length, 0.25 mm inner diameter, 0.25 µm sorbent layer thickness, Restek Corporation, Bellefonte, PA, USA). The injector temperature was set at 240 °C. The oven temperature was maintained isothermally at 40 °C for 1 min, then was raised to 220 °C at a rate of 5 °C min^-1^, and after maintained isothermally for 3 min. Helium at the flow rate of 1.5 mL min^-1^ was used as a carrier gas. Electron ionization spectra were acquired at an electron energy of 70 eV, and the ion source and interface temperatures were held at 230 ºC and 250 ºC, respectively. The volatile compounds released by EPN-infected larvae were identified by comparison of their mass spectral data and retention indexes with the corresponding data available from the NIST version 2.0 mass spectra search program (National Institute of Standards and Technology, USA) and those of synthetic standard of 1-nonene (96%, Sigma Aldrich, USA). GC-MS was used to identify and quantify 1-nonene in SIM mode. The selected ions for 1-nonene were 43; 55; 56; 69; 70; 83; 84; and 126. The quantification of 1-nonene from the sample was carried out by the external calibration curve within the range from 5 to 50 ng (*r*  > 0.99) of this selected compound.

### Statistical analysis

The choice of *S. kraussei* between stimulus (A) and control (B) was calculated as a percentage. IJs falling into both scoring circles (A and B) mentioned above were considered 100%. The percentage of IJs in the A or B circle was calculated by dividing the number of nematodes in each of these circles by the total number of IJs that participated in the assay: A/(A + B) × 100% and B/(A + B) × 100%, respectively. Microsoft Excel was applied to process the data and plot the graphs. Statistical significance (*p* ≤ 0.05) between nematode choices was calculated using the Wilcoxon signed-rank test for the percentage values (PAST 4.03 software). Differences in the emissions of 1-nonene from EPN-infected *G. mellonella* cadavers were calculated using the software Statistica 6.0 (StatSoft, Tulsa, OK, USA). To compare the compound abundance between EPN species, non-parametric Mann-Whitney U test (*p* ≤ 0.05) was used. ANOVA was applied to evaluate emission dynamics within species, followed by Tukey’s HSD test (p ≤ 0.05).

## Results

### Behavioral condition of EPN

**Fig 2:**
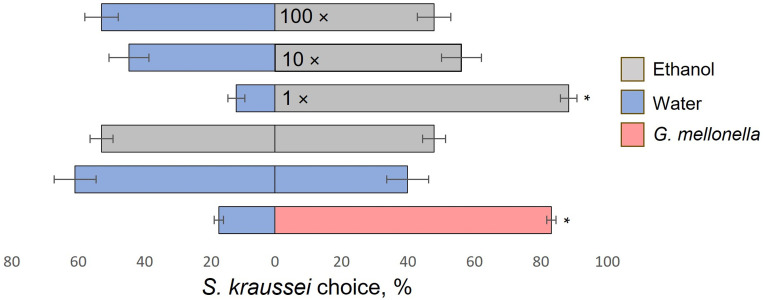
Response of *Steinernema kraussei* infective juveniles (IJs) to different concentrations of ethanol. Percentages (mean ± SEM) represent the ratio of IJs choosing either stimulus or control scoring circles. The supernatant of crushed *Galleria mellonella* larva *vs.* water, water *vs.* water, and ethanol *vs*. ethanol served as controls. Statistically significant differences were assessed using the Wilcoxon signed-rank test, * *p* < 0.001 (suplementary statistical values are provided in S1 Table), horizontal bars represent SEM – standard error of the mean. Each ethanol dilution was tested in three Petri dishes simultaneously and replicated six times.

**Fig 3:**
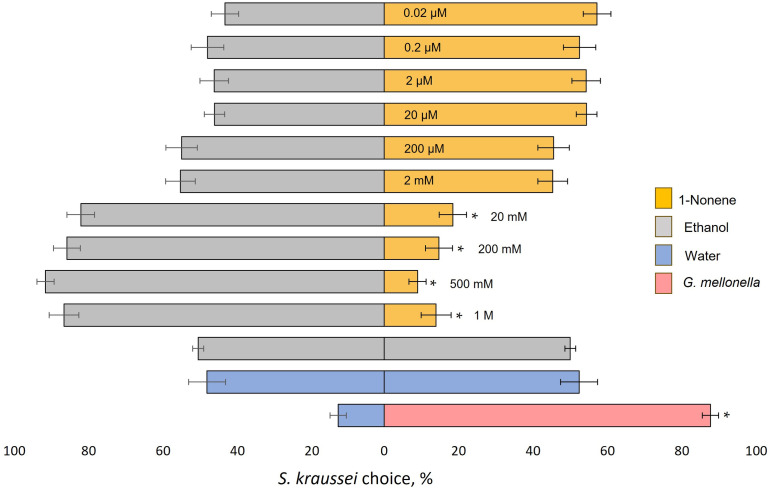
Response of *Steinernema kraussei* infective juveniles (IJs) to different concentrations of 1-nonene. Percentages (mean ± SEM) represent the ratio of IJs choosing either stimulus or control (undiluted ethanol) scoring circles. The supernatant of crushed *Galleria mellonella* larva *vs.* water, water *vs.* water, and ethanol *vs*. ethanol served as controls. Statistically significant differences were assessed using the Wilcoxon signed-rank test, * *p* < 0.001 (suplementary statistical values are provided in S2 Table), horizontal bars represent SEM – standard error of the mean. Each 1-nonene concentration was tested in three Petri dishes simultaneously and replicated six times.

For each of the two-choice behavioral assays, *S. kraussei* IJs’ activity and viability by their response to the positive control supernatant of crushed *G. mellonella* larva *vs.* water, and neutral control – water *vs.* water was evaluated. IJs were strongly attracted to *G. mellonella* – over 80% of the nematodes chose the larvae over water (*p* < 0.001, *W* = 171) (Figs 2 and 3; S1 and S2 Tables). In the water *vs.* water assay, *S. kraussei* chose both Petri dish sides equally, and no statistically significant difference was recorded (Figs 2 and 3; S1 and S2 Tables). All batches of nematodes met both behavioral conditions and were used further for the assay. In the ethanol *vs.* ethanol control assay IJs were distributed equally, and no statistically significant differences were recorded (Figs 2 and 3; S1 and S2 Tables).

### Response of *S. kraussei* to ethanol

Ethanol was strongly attractive to IJs of *S. kraussei*. When undiluted ethanol was applied to the stimulus presentation point, the response percentage was similar to that of *G. mellonella* larvae: 88.6% and 82.2%, respectively (*p* < 0.001, *W* = 171) (Fig 2; S1 Table). Attractivity of 10 times and 100 times diluted ethanol evoked no statistically significant differences *vs.* water control (Fig 2; S1 Table). In summary, ethanol, which further was used as a solvent for 1-nonene presentation, was attractive to *S. kraussei* IJs, but only at the highest concentration.

### Response of *S. kraussei* to 1-nonene

The highest concentrations of 1-nonene (1 M, 500 mM, 200 mM, and 20 mM) were strongly repellent to *S. kraussei* IJs as over 80% of the nematodes chose the ethanol control side of the dish (*p* < 0.001, *W* = 136; 171; 170, and 168, respectivelly) (Fig 3; S2 Table). Lower concentrations (2 mM, 200 µM, 20 µM, 2 µM, 0.2 µM ,0.02 µM) of 1-nonene were neither repellent nor attractive to *S. kraussei* IJs as no statistical differences were observed between the EPN response to the stimulus and the control. In summary, only the highest concentrations of 1-nonene (from 20 mM to 1 M) were repellent to *S. kraussei* IJs.

### 1-Nonene emission from EPN-infected larval cadavers

**Fig 4:**
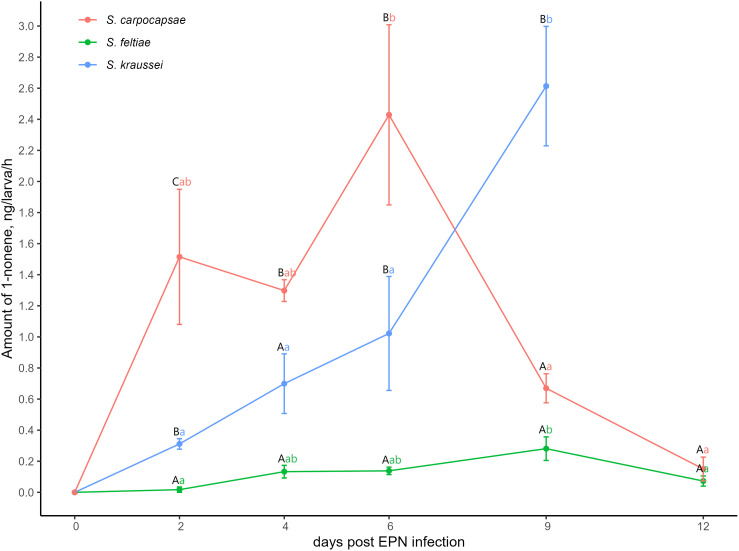
Dynamics of 1-nonene emission from *Steinernema kraussei*, *S. carpocapsae,* or S*. feltiae*-infected *Galleria mellonella* larval cadavers. Statistically significant differences in emissions over time between EPN species are indicated by different uppercase letters, within EPN species are denoted by lowercase letters. Vertical bars indicate standard error. Statistical analysis was performed using the non-parametrical Mann-Whitney U test (*p* ≤ 0.05) and ANOVA followed by Tukey’s HSD test (*p* ≤ 0.05) (supplementary statistical values are provided in S3 and S4 Tables). Three to five volatile samples for each time point and for each species were collected.

1-Nonene emissions were collected from *G. mellonella* larvae infected by either *S. kraussei*, *S. carpocapsae*, or *S. feltiae* at various intervals for 12 days post-infection (Fig 4). Differences in 1-nonene emission between larvae infected by any of the three EPN species were revealed. Overall, 1-nonene emissions were the lowest from larvae infected by *S. feltiae* through the observation period compared to those infected by any of the other two EPN species from the second to the ninth-day post-infection (*p* ≤ 0.05) (Fig 4; S3 and S4 Tables). Moreover, 1-nonene emissions on the second-day post-infection statistically significantly differed among all infection groups. Larvae infected *S. kraussei* released 15 times more 1-nonene (0.31 ± 0.03 ng/larva/h) than those infected with *S. feltiae* (0.02 ± 0.02 ng/larva/h), while larvae infected with *S. carpocapsae* released 76 times more 1-nonene (1.52 ± 0.43 ng/larva/h) than those infected with *S. feltiae* and 5 times more than those infected with *S. kraussei* (*p* ≤ 0.05) (Fig 4; S3 and S4 Tables). *Steinernema feltiae* infected larvae released 1-nonene consistently from the fourth to the ninth day, with emissions decreasing by the 12^th^ day to the levels observed during the first two days after infection. 1-Nonene emission from the larvae infected by either *S. kraussei* or *S. carpocapsae* has risen rapidly and was high from the second to the sixth-day post-infection with no statistically significant differences for each of the species. On the ninth and 12^th^ day post-infection *S. carpocapsae* infection emission dropped significantly and became similar to those of *S. feltiae*. Unfortunately, we could not measure 1-nonene emission on the 12^th^ day post-infection in *S. kraussei-*infected larvae due to overlap with 2-heptanone release. No 1-nonene was collected from the freeze-killed *G. mellonella* larvae (control) and empty Erlenmeyer flasks.

## Discussion

**Reaction to ethanol**. Until recently, it has been known that ethanol is attractive to two species of EPNs: *Heterhorhaditis bacteriophora* [19], *S. feltiae* [14], but wasn’t attractive to *S. carpocapsae* [14]. Among other groups of nematodes, it is known that one species of free-living nematodes, *Caenorhabditis elegans* is attracted to ethanol as well [20,21]. For *H. bacteriophora* and *C. elegans*, ethanol is a mild attractant, while for *S. feltiae*, ethanol is a highly attractive compound [14,19–21]. In this study, for the first time, we revealed that *S. kraussei* IJs were attracted to ethanol, with the attractivity at high concentration close to the model insect prey, a larva of *G. mellonella.* Moreover, the response of *S. kraussei* to ethanol was similar to that reported for *S. feltiae* [14]. Stressed and decaying plant roots are known to release ethanol as a volatile compound (e.g., [22,23]). Ethanol is also recognized as a kairomonal attractant for some above-ground insect species (e.g., [24,25]). Therefore, it could potentially attract below-ground insect herbivores and guide *S. kraussei* to locations with prey, where insects are damaging plant roots. Hence, following the classification of semiochemicals, ethanol might be attributed to apneumones – environmental chemical signals emitted from dead organisms (decayed roots), which is beneficial to the receiver (EPN *S. kraussei*) [26].

**Reaction to 1-nonene.** Among the compounds released by EPN-infected insect cadavers that are behaviorally active to EPN IJs are known as repellents: 3-methyl-2-buten-1-ol (prenol), 3-hydroxy-2-butanone (AMC) [27,28], dimethyl disulfide (DMDS) [10], and attractants: butylated hydroxytoluene (BTH) [29], and 1-nonene [14]. It is known that *S. feltiae* IJs are attracted by low and repelled by high concentrations of 1-nonene, meanwhile, *S. carpocapsae* is attracted by high concentrations only [14]. Also, recently it was revealed that 1-nonene released by *Pseudomonas lurida* bacteria is a weak attractant to another group of nematodes a free-living nematode *C. elegans* [30]. For the first time, we demonstrated that high concentrations of the EPN-infected insect cadaver-released volatile 1-nonene were repellent to *S. kraussei,* and lower ones did not affect this species. Thus, the response of *S. kraussei* to 1-nonene was more similar to IJs of EPN *S. feltiae*. Moreover, we have measured that *G. mellonella* larva infected with *S. kraussei* released an increasing amount of 1-nonene starting from the beginning of the infection which could reach and exceed the threshold for *S. feltiae* behavioural reaction (0.2 µM to 2 µM). However, this indicates that *S. kraussei* can hardly detect the EPN-infected cadaver by the odor of 1-nonene. Other compounds or, more importantly mixture of compounds as in many natural systems, will probably provide more information about the state of infection to *S. kraussei* IJs. Interestingly, as mentioned above, in the previous study, *S. feltiae* was attracted to low concentrations of 1-nonene [14] which could be within the range of concentrations we collected not only from *S. feltiae* infected cadaver (approximately 0.02 µM to 2 µM) but from other infections too (results of the present study). Hence, 1-nonene is an important cue for *S. feltiae* in detecting EPN-infected insect cadavers and should be attributed to apneumones [26].

**The dynamics of 1-nonene release.** In our study for the first time, the dynamics of the volatile organic compound 1-nonene released from insect cadavers infected by the three different EPN species was evaluated. This complements the data on the dynamics of other EPN behaviorally active volatiles released by EPN-infected insect cadavers such as BTH [29], prenol, and AMC [27].

It is known that depending on the EPN species (and its symbiotic bacteria), strain, and the insect host, the pathogenicity/virulence of EPN differs (e.g., [31,32]), as well as the blend of the compounds released, including the ratio of the compounds [9–11]. Hence, based on our results that the emissions of 1-nonene differed for *G. mellonella* larva cadavers infected by the three *Steinernema* species, starting already on the early stage of infection (the second-day significant differences were recorded), we could assume that *S. carpocapsae* is “digesting” the insect faster than *S. kraussei* and even faster than *S. feltiae*. Both the data published in the present paper and that of [9] indicate that 1-nonene emissions differ depending on the insect and nematode species combination.

**Source of 1-nonene**. Since EPN infections in insects involve multiple interacting organisms, volatile emissions may be associated with the insect, the nematode, its symbiotic bacteria, and other bacterial species linked to EPN [33]. Evidence shows that bacteria such as *Comamonas sediminis*, *Pseudomonas monteilii* [34]*, P. lurida* [30], *P. synxantha* [35], *P. fluorescens* [36] produce 1-nonene, classified as alkene. Besides, bacteria are known to produce alkenes when breaking down fatty acids (reviewed in [37]) and through decarboxylation generate 1-alkenes including 1-nonene [38]. Additionally, *P. fluorescens* has been isolated from IJs of *S. feltiae* [39] and *S. carpocapsae* [40,41]. It should be noted that although *G. mellonella* infected with *S. feltiae* and *S. kraussei* carry the same bacterial symbiont, *X. bovienii* [41,42], their 1-nonene emissions differ. *Steinernema kraussei* emission course, however, is more similar to that *S. carpocapsae* infection, where the symbiont is *X. nematophila* [43,44]. This suggests that other bacterial species, beyond the EPN symbionts [33], could contribute to 1-nonene production, particularly in the later stages of infection.

Apart from bacteria producing 1-nonene, there is known that various other microorganisms such as fungi *Aspergillus versicolor* [45], *Penicillium chrysogenum* [45–47], *P. palitans* [46], *P. italicum* [48] release this volatile. Several plant species, e.g., *Arabidopsis thaliana* [49], pear and peach twigs [50] release it into the atmosphere. As already mentioned above, Madagascar ragwort releases 1-nonene in the bouquet of its floral scent [12] as well as *Z. mays* plants when attacked by aphids [13]. Some plants include 1-nonene in their essential oils, i.e., coltsfoot *Tussilago farfara* [51,52], common rue *Ruta graveolens* [53], southern yarrow *Achillea ligustica* [54], swizzle sticks *Senecio anteuphorbium* [55]. 1-Nonene was found also in the epidermal mucus of ray *Gymnura altavela* [56] and in the secretions of abdominal glands of beetles *Bledius spectabilis*, *Platystethus arenarius*, and *Oxytelus piceus* [57].

**Olfaction peculiarities and foraging strategy.** While considerable data is available regarding the responses of various EPN species to plant-derived compounds [58–62], reactions to volatiles emitted by potential insect prey have been less thoroughly investigated. For instance, behavioral responses to 21 insect-derived compounds differed among EPNs with distinct foraging strategies – the cruisers *S. glasseri* and *H. bacteriophora*, the ambushers *S. carpocapsae* and *S. scapterisci*, and the intermediate *S. riobrave* – which displayed different odour-response profiles [63]. Furthermore, no significant differences were observed between the cruiser *S. glaseri* and the intermediate *S. riobrave* in their reactions to volatile compounds, prenol and AMC, released from EPN-infected insect cadavers [27]. To gain a more comprehensive understanding of the link between chemical responses and EPN foraging strategies, further research involving a broader range of compounds and EPN species is needed.

**Summarizing** our results, it is noteworthy that for *S. kraussei*, 1-nonene acts as a repellent, but only at concentrations exceeding those emitted by an EPN-infected cadaver. Our findings, together with those published previously [14], indicate that for *S. carpocapsae*, the attractive concentrations of 1-nonene, and for *S. feltiae*, those that are repellent, significantly exceed the natural levels encountered near an infected cadaver. However, for *S. feltiae,* the concentrations of 1-nonene that elicit attraction remain within the range emitted by an EPN-infected cadaver.

To address whether EPNs’ reactions to chemical compounds released from a decaying insect cadaver are related to their foraging strategy, the available data remain limited. However, based on behavioral responses to environmental compounds that trigger reactions (such as ethanol and 1-nonene), both the cruiser (*S. kraussei*) and intermediate (*S. feltiae*) species exhibit similar behavior, contrasting with the response of the ambusher (*S. carpocapsae*). This suggests that among the three ecological groups of EPNs, two stand out sharply based on their reactions to odorous compounds.

For biological control applications, it is important to consider that EPNs with different olfactory peculiarities and foraging strategies may vary in their effectiveness. More accurate insights into the association between chemical responses and EPN foraging strategies may be obtained by testing a broader range of compounds and EPN species. Further research in this direction would be valuable for advancing our understanding.

## Supporting information

S1 Table Statistical values (Wilcoxon signed-rank test) of *Steinernema kraussei* behavioral assay to ethanol. (DOCX)

S2 Table Statistical values (Wilcoxon signed-rank test) of *Steinernema kraussei* behavioral assay to 1-nonene. (DOCX)

S3 Table Statistical values (Mann-Whitney U test) for comparisons of 1-nonene emissions between *Galleria mellonella* cadavers infected with three different species of entomopathogenic nematodes. (DOCX)

S4 Table Statistical values (ANOVA followed by Tukey’s HSD test) for comparisons of 1-nonene emission dynamics within *Galleria mellonella* cadavers infected with each of three different species of entomopathogenic nematodes. (DOCX)
