# Supplementary material for: Scent of death: Emission and behavioral role of 1-nonene in entomopathogenic nematode Steinernema kraussei
Source: PLoS One. 2025 Jul 28;20(7):e0328628. doi: 10.1371/journal.pone.0328628 (PMC12303281; doi:10.1371/journal.pone.0328628)
Supplement: S1 Table — (DOCX) [file pone.0328628.s001.docx]

**Table S1. Statistical values (Wilcoxon signed-rank test) of *Steinernema kraussei* behavioral assay to ethanol.** Green boxes indicate *p* ≤ 0.05.

| **Treatment** | | **Statistical value** | | | |
| --- | --- | --- | --- | --- | --- |
|  |  | **N** | ***p*** | ***z*** | ***W*** |
| **Control** | *Galleria. mellonella*  Water  Ethanol | 18 | 0.0002  0.2854  0.8788 | 3.7240  1.0683  0.1524 | 171  110  89 |
| **Ethanol** | 1 ×  10 ×  100 × |  | 0.0002  0.2064  0.3161 | 3.7244  1.2634  1.0026 | 171  114.5  108.5 |
