# Supplementary material for: Scent of death: Emission and behavioral role of 1-nonene in entomopathogenic nematode Steinernema kraussei
Source: PLoS One. 2025 Jul 28;20(7):e0328628. doi: 10.1371/journal.pone.0328628 (PMC12303281; doi:10.1371/journal.pone.0328628)
Supplement: S2 Table — (DOCX) [file pone.0328628.s002.docx]

**Table S2.** **Statistical values (Wilcoxon signed-rank test) of *Steinernema kraussei* behavioral assay to 1-nonene.** Green boxes indicate *p* ≤ 0.05.

| **Treatment** | | **Statistical value** | | | |
| --- | --- | --- | --- | --- | --- |
|  |  | **N** | ***p*** | ***z*** | ***W*** |
| **Control** | *Galleria mellonella*  Water  Ethanol | 18 | 0.0002  0.8766  0.6474 | 3.7253  0.1552  0.4574 | 171  71  96 |
| **1-Nonene** | 1 M  500 mM  200 mM  20 mM  2 mM  200 µM  20 µM  2 µM  0.2 µM  0.02 µM |  | 0,0004  0.0002  0.0002  0.0003  0.3719  0.1988  0.0928  0.4080  0.5861  0.1024 | 3.5496  3.7324  3.6818  3.5938  0.8929  1,2849  1.6807  0.8275  0.5444  1.6333 | 136  171  170  168  106  115  112  84  98  123 |
