# Supplementary material for: Scent of death: Emission and behavioral role of 1-nonene in entomopathogenic nematode Steinernema kraussei
Source: PLoS One. 2025 Jul 28;20(7):e0328628. doi: 10.1371/journal.pone.0328628 (PMC12303281; doi:10.1371/journal.pone.0328628)
Supplement: S3 Table — (DOCX) [file pone.0328628.s003.docx]

**Table S3.** **Statistical values (Mann-Whitney U test) for comparisons of 1-nonene emissions between Galleria mellonella cadavers infected with three different species of entomopathogenic nematodes.** Green boxes indicate *p* ≤ 0.05. Species compared: A – *Steinernema feltiae* (N=4); B – *S. kraussei* (N=5), C – *S. carpocapsae* (N=3).

| **Day post-infection** | **Compared species** | ***Z*** | ***p*** |
| --- | --- | --- | --- |
| **2** | A *vs.* B  A *vs.* C  B *vs.* C | -2.4914  -2.2014  -2.2361 | 0.01273  0.02771  0.02535 |
| **4** | A *vs.* B  A *vs.* C  B *vs.* C | -1.9596  -2.1213  -2.2361 | 0.05004  0.03390  0.02535 |
| **6** | A *vs.* B  A *vs.* C  B *vs.* C | -2.4598  -2.1405  -1.6398 | 0.01390  0.03231  0.10105 |
| **9** | A *vs.* B  A *vs.* C  B *vs.* C | -2.4495  -1.7678  2.2361 | 0.01431  0.07710  0.02535 |
| **12** | A *vs.* C | -0.8917 | 0.37246 |
