# Supplementary material for: Scent of death: Emission and behavioral role of 1-nonene in entomopathogenic nematode Steinernema kraussei
Source: PLoS One. 2025 Jul 28;20(7):e0328628. doi: 10.1371/journal.pone.0328628 (PMC12303281; doi:10.1371/journal.pone.0328628)
Supplement: S4 Table — (DOCX) [file pone.0328628.s004.docx]

**Table S4. Statistical values (ANOVA followed by Tukey’s HSD test) for comparisons of 1-nonene emission dynamics within Galleria mellonella cadavers infected with each of three different species of entomopathogenic nematodes.** Green boxes indicate *p* ≤ 0.05. Species: A – *Steinernema feltiae* (N=4); B – *S. kraussei* (N=5), C – *S. carpocapsae* (N=3).

| **Species** | **Days compared** | ***p*** |
| --- | --- | --- |
| **A** | 2 *vs.* 4 | 0.36515 |
|  | 2 *vs.* 6 | 0.32474 |
|  | 2 *vs.* 9 | 0.00496 |
|  | 2 *vs.* 12 | 0.89079 |
|  | 4 *vs.* 6 | 0.99998 |
|  | 4 *vs.* 9 | 0.16543 |
|  | 4 *vs.*12 | 0.85926 |
|  | 6 *vs.* 9 | 0.19015 |
|  | 6 *vs.* 12 | 0.81997 |
|  | 9 *vs.* 12 | 0.02814 |
| **B** | 2 *vs.* 4 | 0.76839 |
|  | 2 *vs.* 6 | 0.31957 |
|  | 2 *vs.* 9 | 0.00032 |
|  | 4 *vs.* 6 | 0.85005 |
|  | 4 *vs.* 9 | 0.00118 |
|  | 6 *vs.* 9 | 0.00547 |
| **C** | 2 *vs.* 4 | 0.98898 |
|  | 2 *vs.* 6 | 0.34994 |
|  | 2 *vs.* 9 | 0.41829 |
|  | 2 *vs.*12 | 0.08807 |
|  | 4 *vs.* 6 | 0.18620 |
|  | 4 *vs.* 9 | 0.67141 |
|  | 4 *vs.* 12 | 0.17649 |
|  | 6 *vs.* 9 | 0.02392 |
|  | 6 *vs.* 12 | 0.00459 |
|  | 9 *vs.* 12 | 0.79630 |
